# Social isolation enhances cued-reinstatement of sucrose and nicotine seeking, but this is reversed by a return to social housing

**DOI:** 10.1038/s41598-021-81966-2

**Published:** 2021-01-28

**Authors:** Natalie A. Mastrogiovanni, Alice K. Wheeler, Kelly J. Clemens

**Affiliations:** School of Psychology, University of New South Wales, Sydney, Australia

**Keywords:** Reward, Extinction, Habituation, Operant learning, Addiction, Stress and resilience

## Abstract

Physical or perceived (i.e. loneliness) social isolation is increasing in Western cultures. Unfortunately, social isolation is associated with a range of negative physical and mental health outcomes, including increased incidence of obesity and smoking. Here we monitored the impact of social isolation on a range of physical measures, and then tested whether social isolation in adult rats changes how reward-related stimuli motivate sucrose- or nicotine-seeking. Socially isolated rats showed elevated baseline CORT, gained significantly less weight across the study, were more active in response to a novel or familiar environment. Isolated rats also acquired nose-poking for a food pellet more rapidly, and showed increased susceptibility to cue-, but not reward-induced reinstatement. Notably, these effects are partially mitigated by a return to group housing, suggesting that they are not necessarily permanent, and that a return to a social setting can quickly reverse any deficits or changes associated with social isolation. This study advances our understanding of altered reward-processing in socially isolated individuals and reiterates the importance of socialisation in the treatment of disorders such as overeating and addiction.

## Introduction

The Australian Psychological Society (APS) reports that one in four Australians feel lonely and over half of the population feel that they lack valuable social connection^1^. Whether objective or perceived (i.e. loneliness), the consequences of prolonged social isolation are significant. Social isolation is linked to severe negative health implications including increased risk of heart disease^2^, cancer^3^ and obesity^4^, culminating in reduced life expectancy^5,6^. Social isolation also comes with significant risk of mental health and neuropsychiatric disorders, including chronic anxiety and depression^7,8^. Alongside this complex aetiology, social isolation has been linked to the increased prevalence of substance use disorders across a range of drug types^7^, where social isolation both predicts drug abuse, and drug abuse occurs as a consequence of social isolation^9–11^. Unfortunately, when socially isolated individuals wish to moderate or quit drug-intake, quitting is more difficult and less successful^12,13^, limiting the likelihood of a long lasting recovery.

The link between social isolation and addiction is complex and influenced by an array of factors including socioeconomic status, social anxiety and stress^14^, however there is also some evidence to suggest socially isolated individuals encode information in their environment differently than non-isolated people. It is well established that anxious individuals are hypervigilant to stimuli in their environment, including those perceived as threatening^15^, but also those of a positive valence^16^. A similar pattern has been observed in socially isolated or lonely individuals, who demonstrate enhanced perception of threat^17–19^ and attentional bias towards threatening stimuli^20^. This bias may also extend to positive social stimuli, where loneliness changes how pleasant pictures are perceived and encoded^21^. Considering how potently drug-associated stimuli (or cues) rapidly acquire incentive value and come to exert control over reward seeking behaviour^22^, increased vigilance to drug-associated cues may be a strong contributing factor to the persistence of drug-seeking in isolated individuals.

Further support for a link between social isolation and heightened cue-reward processing comes from animal studies. Consistent with the human data, socially isolated or individually housed rats display an anxiogenic profile on physiological^23,24^ and behavioural^25,26^ measures of stress and anxiety. Socially isolated rats also show altered sensitivity to reward-related cues, including increased approach^27^ and responding for a cue previously paired with sucrose^28^, as well as potentiated cue-induced reinstatement of responding for sucrose^29^. This suggests increased incentive value of cues associated with highly palatable foods.

Socially isolated rats also show an altered response to drugs of abuse, including facilitated acquisition of nicotine, cocaine and heroin^30–32^ self-administration, which has been linked to interactions with the hypothalamic–pituitary–adrenal (HPA) axis^33^. However, social isolation may also change sensitivity towards and in response to drug-paired cues, with evidence of enhanced reinstatement to methamphetamine-paired cues in mice^34^ and cocaine-paired cues in rats^35^. Together these studies suggest that social isolation alters the way rats react to cues in their environment that either predict or are associated with both highly palatable food and drug rewards. However, the majority of existing studies assess the impact of isolation applied in juvenile or adolescent stages of development. Whether the experience of social isolation in adulthood similarly contributes to how rats acquire and relapse to drug-seeking, as well the potential reversal of such effects by social re-integration, is not clear.

In the current study, we first examined the impact of social isolation on responding for both a highly palatable food reward (sucrose pellet) and a drug reward (nicotine), and the cues that were associated with their intake. We used a food reward to determine if any alterations in cue-associated reward processing were unique to drug rewards or were common across both reward types. We selected nicotine due to its known properties as both a reinforcer and reinforcement enhancer^42^, and evidence of high rates of smoking in the socially isolated^11,12^.

The second aim is to assess whether the consequences of social isolation can be reversed with a return to group housing. One approach to the treatment of social isolation is to reengage with social contexts^36^, however the consequences for later drug seeking are not clear. By switching rats from social isolation to group housing (and vice versa) mid-experiment, we can also interrogate whether it is the encoding of cue-drug associations that is altered in socially isolated rats, or whether it is the retrieval of this information at test that is important.

## Methods

### Subjects and housing

Male Long-Evans rats (231–395 g) obtained from our in-house breeding facility (University of New South Wales, Randwick, Australia) were initially housed four per cage in large tubs (58 cm × 35 cm × 27 cm) on a 12 h reverse dark/light cycle (lights on at 1900 h). Food was initially available ad libitum and then restricted to 22–26 g/rat/day 2–3 days prior to the initiation of instrumental training.

Following a seven-day acclimation period rats were evenly allocated (based on body weight) to remain in existing group housing, or to be isolated for 20 days prior to instrumental training. Rats remained in these housing conditions for the entire duration of the experiment unless otherwise indicated (Expt. 3 and 4). Rats in the socially isolated treatment condition were individually housed in medium sized cages (60 cm × 26 cm × 30 cm) that included a mezzanine floor. Both housing conditions contained enrichment in the form of large wooden chew blocks and red Perspex tunnels.

All procedures were approved by the UNSW Animal Ethics Committee and performed in accordance with the Australian Code for the Care and Use of Animals for Scientific Purposes (8th ed, 2013) and the ARRIVE guidelines^37^.

### Drugs

(−)-Nicotine hydrogen tartrate (#SML1236; Sigma-Aldrich, St Louis, MO, USA) was dissolved in sterile sodium chloride solution (0.9%) and administered intravenously at a dose of 0.03 mg/kg/100µL infusion or sub-cutaneously (for reinstatement) at a dose of 0.3 mg/kg. All concentrations refer to the nicotine base.

### Intravenous catheter surgery

For experiments 2 and 4, prior to the beginning of testing all rats underwent surgery for the implantation of a chronic intravenous catheter and recovered for 7 days prior to testing and as described previously^38^.

### Apparatus

All instrumental training was conducted in standard tall self-administration chambers (Med Associated, VT, USA) equipped with two nose-poke holes either side of a food magazine. For instrumental conditioning with a food reward, a pellet dispenser was located above the magazine, external to the chamber. For self-administration, the intravenous catheter back mount on each rat was connected to plastic tubing inside a metal spring connector that attached to a fluid swivel assembly and weighted arm at the top of the cage. The tubing then exited the sound attenuation chamber and attached to a 20 mL syringe containing nicotine housed in a syringe pump (see^38^ for additional details).

### Procedures

**Figure 1:**
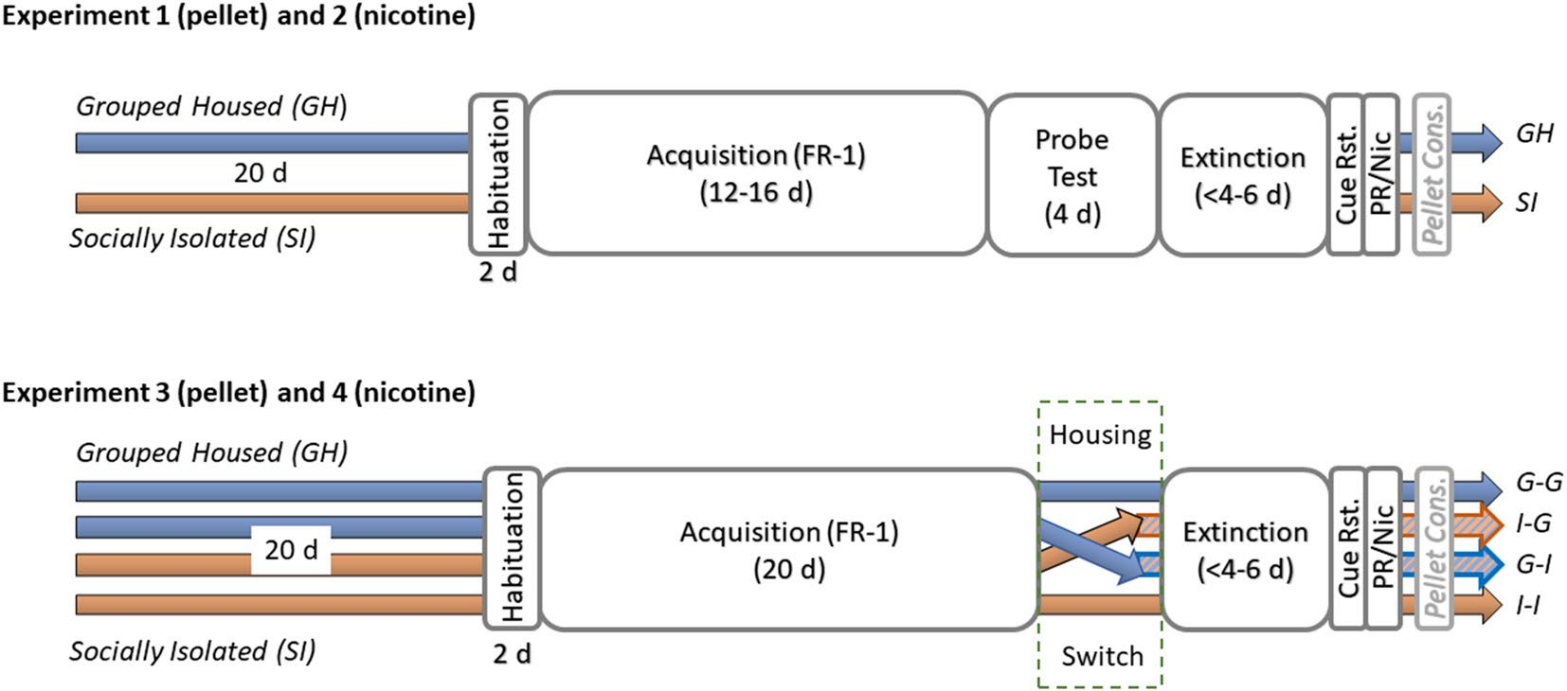
Experimental timeline of the study. Four experiments were conducted separately: experiments 1 and 2 included a probe test (cue only or reward only) and experiments 3 and 4 included a change of housing conditions after acquisition. The number of days spent in each phase of each study are indicated, with cue reinstatement (Cue Rst.), progressive ratio test (for Experiments 1 and 3) and nicotine reinstatement tests (for Experiments 2 and 4) were conducted on single consecutive days. The pellet consumption test (Pellet cons.) was performed for rats in Experiments 1 and 3 only, as these rats received a pellet reward. GH and G–G indicates rats that remained group housed throughout the study; I–G indicates rats that were initially socially isolated and switched to group housing following initial instrumental conditioning; G–I indicates rats that were initially group housed and switched to social isolation following initial instrumental conditioning; SI and I–I indicates rats that remained socially isolated for the duration of the study.

The overall experimental procedure is shown in Fig. 1. Rats in Experiment 1 and 2 remained in the same housing condition for 20 days prior to training and for the duration of the study: Group housed (GH) and socially isolated (SI).

For Experiments 3 and 4, half of the rats switched housing condition after initial training (acquisition) and for the remainder of the experiment (extinction and reinstatement), resulting in four treatment groups reflecting housing at the two stages: Group–Group (G–G), Group-Isolated (G–I), Isolated–Group (I–G) and Isolated–Isolated (I–I).

#### Experiment 1: The effect of social isolation on the acquisition and reinstatement of responding for a sucrose pellet

Rats were initially housed in groups of 4 (n = 16) or single housed (n = 16), before being evenly split (based on active responses and rewards earned made across the last 3 days of acquisition) into group housed cue-only (n = 8), group housed pellet only (n = 8), single housed cue-only (n = 8) or single housed pellet only (n = 8) conditions for the probe test sessions.

*Instrumental Conditioning:* Training for all rats commenced with 2 × daily 30 min habituation sessions where rats were placed in the chambers with nose-poke holes covered and five sucrose pellets in the magazine. The house-light remained on and locomotor activity was recorded.

The following day, 12 days of operant conditioning commenced where a single response on the active nose-poke resulted in the delivery of a food pellet into the magazine, illumination of a light emitting diode (LED) stimulus inside the nose-poke and offset of the house-light for 20 s. Sessions continued for 30 min or until 30 pellets had been obtained.

*Probe Trials:* To assess the source of motivation to respond, we tested the ability of the food or cue alone to maintain responding across 4 days of instrumental training. Rats were equally divided into two groups: The first group (pellet-only) received response-contingent pellet delivery, but no visual cues were presented and there was no maximum number of pellets; The second group (cue-only) received response-contingent visual cue presentation, but no pellet was delivered. Following this testing, rats then underwent an additional 4–5 days of training under baseline conditions (reacquisition).

*Extinction:* All rats underwent extinction training on four consecutive days. During these 30 min sessions, the house-light remained on and nose-poke responses were of no consequence.

*Reinstatement:* Rats underwent a single 30 min cue-reinstatement test where the response contingent cue was reintroduced under the same conditions as original training, but no sucrose pellet was delivered.

The following day, rats underwent a progressive ratio (PR) test to measure motivation for the sucrose pellet, where the pellet was available according to the following increase in ratio requirement: 1, 2, 4, 6, 9, 12, 15, 20, 25, 32, 40, 50, 62, 77, 95, 118, 145. The session ended if no responses were made for 30 min, or after one hour had elapsed. This was imposed to measure motivation under conditions that would otherwise produce very high rates of responding and would ultimately be affected by satiety.

*Pellet Consumption:* The following day, rats were transferred to novel individual cages and given free access to 300 sucrose pellets for 30 min. The number of pellets consumed across this period was calculated and adjusted to account for body weight.

#### Experiment 2: The effect of social isolation on the acquisition and reinstatement of responding for intravenous nicotine

Rats were initially housed in groups of 4 (n = 16) or single housed (n = 16), before being evenly split (based on active responses and rewards obtained across the last 3 days of acquisition) into group housed cue-only (n = 8), group housed pellet only (n = 8), single housed cue-only (n = 8) or single housed pellet only (n = 8) conditions for the probe test sessions.

*Serum Corticosterone (CORT):* Venous blood was sampled from catheterised rats in Experiment 2 prior to the beginning of training. This time point was selected as it followed sustained social isolation (20 d), all rats had patent catheters (permitting the withdrawal of 200 µL of blood with minimal stress or restraint of the rat) and it reflected baseline CORT levels of rats prior to entering into instrumental training. Samples were incubated at room temperature for 30 min, centrifuged at 1000×*g* for 10 min and clot removed. Serum was then transferred into clean tubes and stored at − 80 °C until processing. Serum CORT was determined using a competitive ELISA kit (#ab108821; Abcam, MA, USA) and according to the manufacturer’s instructions.

*Instrumental Training:* Training for nicotine self-administration progressed largely as described above for sucrose pellets, with the following differences:

Habituation sessions lasted for 60 min. A nose-poke resulted in the infusion of nicotine (0.03 mg/kg/infusion) delivered across 3 s, sessions were 60 min long and were conducted across 16 days.

Extinction training sessions were 60 min long and continued for a minimum of 6 days and maximum of 10 days, or until responding was < 30% of that recorded during training.

Reward-primed reinstatement involved administration of a single sub-cutaneous injection of nicotine (0.3 mg/kg) immediately prior to the test session.

#### Experiment 3 and 4: The effect of social isolation on acquisition versus reinstatement of responding for a sucrose pellet (Expt. 3) or intravenous nicotine (Expt. 4)

Experiments 3 and 4 assessed whether the observed differences at cue-induced reinstatement in Experiment 1 and 2 were due to the impact of social isolation on either the acquisition of the cue-reward association, or on the expression or recall of these associations at test.

Experiments 3 and 4 were identical to Experiments 1 and 2, respectively (initially 32 rats per experiment, GH = 16, SI = 16) with the exception that there was no probe testing. After acquisition half of the group housed animals were socially isolated (Group G–I) and half of the isolated animals were rehoused in pairs with a conspecific from their original home cage (Group I–G), resulting in 8 rats/group for continued group housing (G–G), group housed and then isolated (G–I), isolated and then group housed (I–G) and continued social isolation (I–I). Rats were closely monitored when moving from SI to GH (i.e. group I–G) and if fighting was observed, these animals were separated. This happened with one pair from Expt. 3 resulting in 6 rats in the I–G group and 10 in group I–I.

### Statistical analysis

Data were analysed (IBM SPSS Statistics 26) using 2-tailed t-tests and full factorial General Linear Model Univariate or Repeated Measures ANOVAs with alpha set at 0.05. For experiments 1 and 2, there were two between subjects’ factors: the first was housing condition (group vs single) and the second was probe trial (cue only or reward only). For experiments 3 and 4, there were two between subjects factors: the first was housing condition at time 1 (group vs single) and the second was housing condition at time 2 (group vs single). When analysing nose-poke data there was a within subjects’ factors of nose poke (active or inactive). When analysing data gathered across time (e.g. body weight, acquisition, extinction, reinstatement), there was an additional within subjects’ factor of day. For reasons of clarity and brevity, only significant main effects or interactions are reported. Partial Eta Squared is included as an estimate of effect size^39^ on all ANOVAs, where values of 0.10, 0.25, and 0.50 are considered estimates of small, medium and large effect sizes respectively.

For experiments 1 and 3, all rats successfully acquired the instrumental response and completed the entire study. All data (n = 8/sub-group) were included in the analysis. For experiment 2, 3 rats did not complete acquisition (2 × GH, 1 × SI), and 2 did not complete the whole study (2 × SI) due to catheter failure. For Experiment 4, one rat (group G–G) was excluded due to loss of catheter patency.

## Results

### Experiment 1: The effect of social isolation on the acquisition and reinstatement of responding for a sucrose pellet

#### Body weight

**Figure 2:**
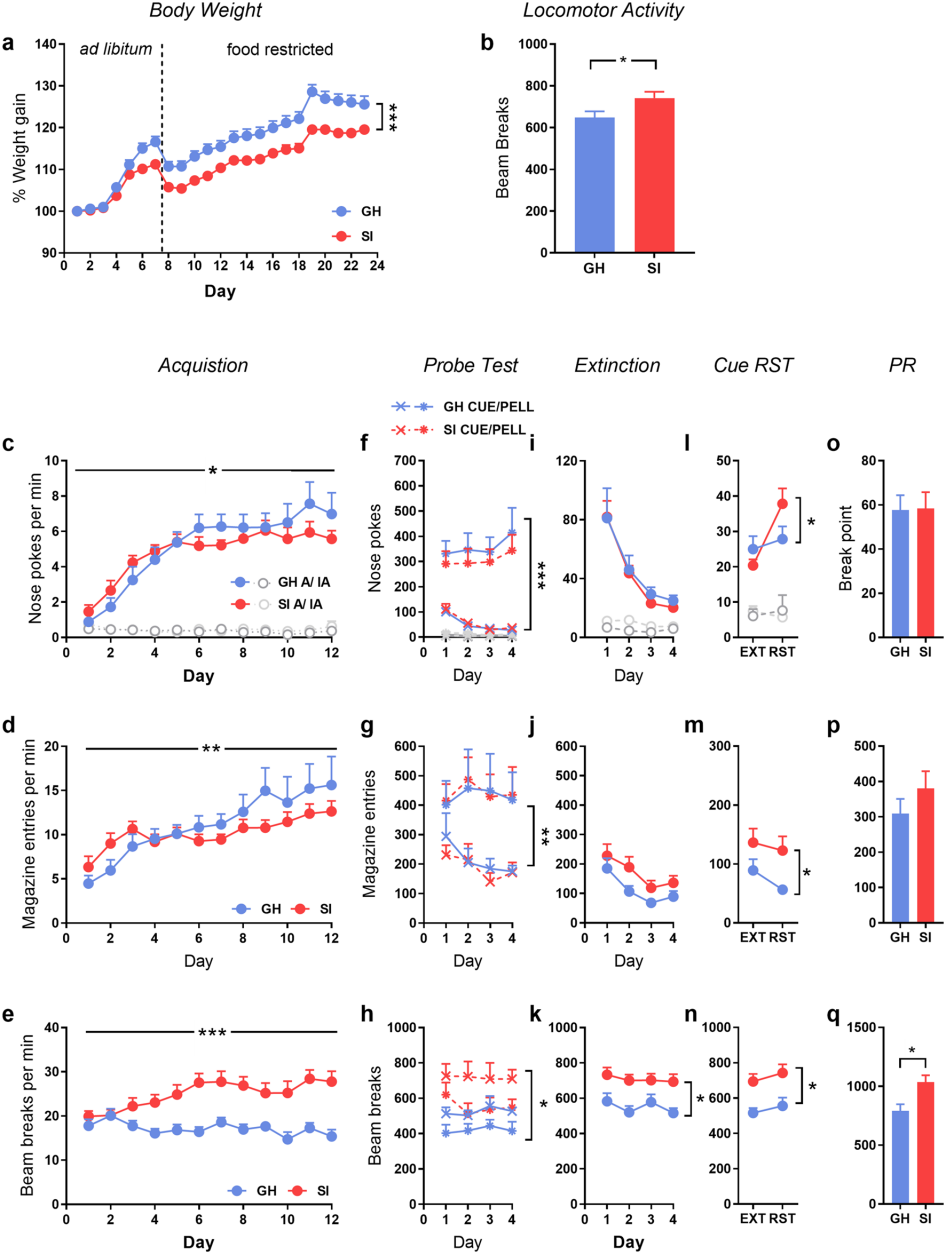
Experiment 1: The effect of social isolation on the acquisition and reinstatement of responding for a sucrose pellet. (**a**) Percentage weight gain across the entire study, including when food was freely available (ad libitum) and following restriction prior to operant training (food restricted). (**b**) Spontaneous locomotor activity during habituation to the instrumental chambers. Active (colour) and inactive (grey) nose-pokes, magazine entries and locomotor activity across acquisition (**c**–**e**), the probe test trial (**f–h**), extinction training (**i**–**k**), cue-reinstatement (**l–n**) and the progressive ratio (PR) test of motivation for the food reward (**o**–**q**). *GH* group housed, *SI* socially isolated. Asterisks indicate main effect or interaction involving housing condition where *p < 0.05, **p < 0.01 or ***p < 0.001. n = 16/group except 8/group for the probe test.

As illustrated in Fig. 2a, socially isolated rats gained less weight across the duration of the study in comparison to group housed rats (main effect of housing: F(1,30) = 13.23, *p* < 0.001, $\eta_{\rho }^{2}$ = 0.31, day x housing: F(22,660) = 8.347, *p* < 0.001, $\eta_{\rho }^{2}$ = 0.22).

#### Acquisition of instrumental conditioning

Socially isolated rats showed greater spontaneous locomotor activity, as indicated by a higher number of infrared beam breaks across habituation to the operant chamber (Fig. 2b; t(30) = 2.20, *p* < 0.05).

Once training commenced, all rats rapidly learned to nose-poke in order to receive a sucrose pellet, as indicated by an increasing preference for the active nose-poke (Fig. 2c; F(1,30) = 299.152, *p* < 0.001, $\eta_{\rho }^{2}$ = 0.91) that increased across days (F(11,330) = 19.20, *p* < 0.001, $\eta_{\rho }^{2}$ = 0.39). However, the pattern of acquisition (responses per minute) differed between groups (nose-poke x day x housing interaction: F(11,330) = 2.784, *p* < 0.05, $\eta_{\rho }^{2}$ = 0.85), where SI rats initially acquired the nose-poke response more rapidly followed by continued responding at a slightly lower level overall.

The rate of magazine entries reflected the difference in nose-pokes (Fig. 2d; day x housing interaction: F(11,330) = 3.315, *p* < 0.001, $\eta_{\rho }^{2}$ = 0.10). Consistent with the habituation data, SI rats were overall more active than GH rats (Fig. 2e; F(1,30) = 17.190, *p* < 0.001, $\eta_{\rho }^{2}$ = 0.28), with this difference increasing across training days (day x housing; F(11,330) = 5.76, *p* < 0.001; $\eta_{\rho }^{2}$ = 0.08). This pattern of activity confirms that the observed differences in responding and magazine entries are not due to group differences in activity, as by the end of training GH rats are responding more and making more magazine entries, yet have lower overall activity.

#### Probe trial for pellet- or cue-maintained responding

As shown in Fig. 2f, nose-poke responding was largely driven by responses for the pellet, as removal of the response-contingent cues resulted in a sustained high level of responding for the pellet (pellet only), whereas removal of pellet delivery, but retention of the cue-light (cue only) sustained low levels of responding only (main effect of test condition: F(1,28) = 37.53, *p* < 0.001, $\eta_{\rho }^{2}$ = 0.57). Active and inactive responding for the cue only, or pellet only was not influenced by housing condition (*p*s < 0.05).

Not surprisingly magazine entries were also much lower in the cue-only groups compared to pellet-only conditions (Fig. 2g; F(1,28) = 11.672, *p* < 0.01, $\eta_{\rho }^{2}$ = 0.30), and this was not impacted by housing condition.

The group differences in locomotor activity were maintained across the test phase, with SI rats again more active than GH (Fig. 2h; main effect of housing: F(1,28) = 11.55, *p* < 0.01, $\eta_{\rho }^{2}$ = 0.30), and rats in the cue-only condition overall more active than the pellet only rats (F(1,28) = 7.75, *p* < 0.05, $\eta_{\rho }^{2}$ = 0.22), again suggesting that observed differences in responding were not due to differences in locomotor activity.

#### Extinction and reinstatement

Nose-poke responding across extinction did not differ between housing conditions across extinction training (Fs > 1) or on the last extinction day (Fig. 2i). Magazine entries were not different from each other across this period (Fig. 2j; p = 0.091), although SI rats again showed greater levels of locomotor activity (Fig. 2k; F(1,30) = 17.596, *p* < 0.001, $\eta_{\rho }^{2}$ = 0.40) compared to the group housed rats.

Overall, rats reinstated responding on the active nose-poke following the return of the response-contingent cue (Fig. 2l; main effect of test vs extinction: F(1,28) = 6.16, *p* < 0.05, $\eta_{\rho }^{2}$ = 0.18; main effect of nose-poke: F(1,28) = 8.29, *p* < 0.01, $\eta_{\rho }^{2}$ = 0.78) and to a greater extent in SI rats (nose-poke x test x housing interaction: (F(1,28) = 6.37, *p* < 0.05, $\eta_{\rho }^{2}$ = 0.19). Responding was overall higher in rats that had been in the pellet only probe condition (F(1,28) = 6.72, *p* < 0.05, $\eta_{\rho }^{2}$ = 0.19), however this did not interact with housing condition and therefore is not included as a factor for further analysis. Magazine entries were overall higher across both extinction and reinstatement in the SI rats (Fig. 2m; F(1,28) = 5.216, *p* < 0.05, $\eta_{\rho }^{2}$ = 0.16), as was locomotor activity (Fig. 2n; F(1,28) = 16.17, *p*0.01, $\eta_{\rho }^{2}$ = 0.37), suggesting that the pattern of nose-poke responding was not driven by changes in general activity in the SI group.

There were no group differences in breakpoint on a subsequent PR test for motivation to obtain the sucrose pellet (Fig. 2o). Magazine entries across this session were similar (Fig. 2p; *p* = 0.275) although locomotor activity was again higher in the SI group compared to GH rats (Fig. 2q; F(1,28) = 9.27, *p* < 0.01, $\eta_{\rho }^{2}$ = 0.24). There were no group differences in a subsequent pellet consumption test (% consumption/body weight: GH = 3.58 ± 0.25; SI = 3.48 ± 0.13), suggesting that the effects observed across the study are not due to altered motivation or appetite for the sucrose pellet.

### Experiment 2: The effect of social isolation on the acquisition and reinstatement of responding for intravenous nicotine

#### Body weight

**Figure 3:**
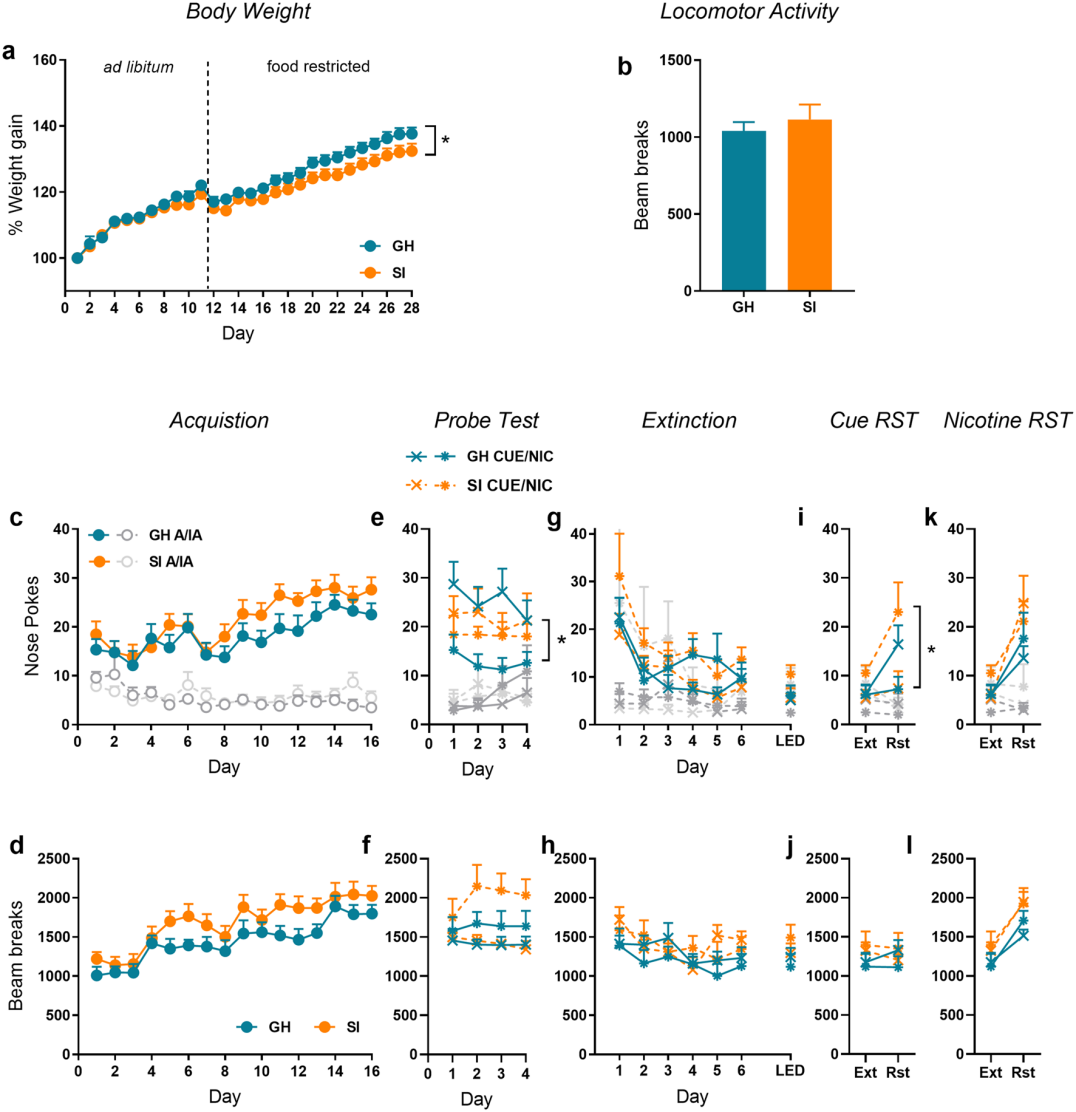
Experiment 2: The effect of social isolation on the acquisition and reinstatement of responding for intravenous nicotine. (**a**) Percentage weight gain across the entire study, including when food was freely available (ad libitum) and following restriction prior to operant training (food restricted). (**b**) Spontaneous locomotor activity during habituation to the instrumental chambers. Active (colour) and inactive (grey) nose-pokes and locomotor activity across acquisition (**c**, **d**), the probe test trial (**e**, **f**), extinction training (**g**, **h**), cue-reinstatement (**i**, **j**) and nicotine reinstatement (**k**, **l**). *GH* group housed, *SI* socially isolated, *LED* Last extinction day, *EXT* extinction, *RST* reinstatement. Asterisks indicate main effect or interaction involving housing condition where *p < 0.05, **p < 0.01 or ***p < 0.001. n = 16/group except 8/group for the probe test.

Consistent with Experiment 1, socially isolated rats gained significantly less weight across the duration of the study (Fig. 3a; day x housing interaction: F(28,840) = 2.902, *p* < 0.001, $\eta_{\rho }^{2}$ = 0.08).

#### Serum corticosterone

Serum corticosterone levels were significantly elevated in socially isolated rats following 20 days of isolation and immediately prior to self-administration training (group housed: 642.63 pg/mL ± 176.27; socially isolated: 1329.44 pg/mL ± 221.96; t(25) = 2.246, *p* < 0.05), confirming that social isolation produces a chronic mild stress.

#### Acquisition of intravenous nicotine self-administration

Locomotor activity across habituation to the operant chamber did not differ between groups (Fig. 3b).

All rats rapidly acquired the self-administration response as indicated by a greater number of responses on the active nose poke that increased across days (Fig. 3c; nose poke: F(1,26) = 93.99, *p* < 0.001, $\eta_{\rho }^{2}$ = 0.78; day: F(15,390) = 6.71, *p* < 0.001, $\eta_{\rho }^{2}$ = 0.21; nose poke x day: F(15,390) = 8.120, *p* < 0.001, $\eta_{\rho }^{2}$ = 0.24). However, the rate of acquisition was not impacted by housing condition.

Locomotor activity across acquisition did not differ between groups (Fig. 3d).

#### Probe trial for nicotine- or cue-maintained responding

Across the test phase, a nose poke by housing by test condition (Fig. 3e; F(1,22) = 4.64, *p* < 0.05, $\eta_{\rho }^{2}$ = 0.22) shows that responding on the active nose-poke differed depending both on test (i.e. cue-only or pellet-only) and housing condition. Follow-up analysis on each group separately shows that whereas rats in the SI condition respond equally for nicotine alone or the cues alone (F < 1), rats in the GH condition respond more for cues than nicotine (nose-poke by test interaction: F(1,11) = 12.30, *p* < 0.01, $\eta_{\rho }^{2}$ = 0.55).

Locomotor activity across this probe period was significantly higher overall in animals responding for the cue only (Fig. 3f; F(1,23) = 5.541, *p* < 0.05, $\eta_{\rho }^{2}$ = 0.18), however this was not impacted by housing condition. The pattern of activity did not follow the same pattern as nose-poke responding, indicating that the group differences in responding were not due to differences in locomotor activity.

#### Extinction and reinstatement

There were no group differences due to housing on the number of responses made across the first 6 days of extinction (Fig. 3g), the number of days taken to reach the extinction criterion (GH = 8.71 ± 0.41; SI = 7.71 ± 0.70), or responses on the last day of extinction. Housing condition did not affect locomotor activity across extinction training (Fig. 3h).

The previously allocated probe trial test condition had a lasting impact on reinstatement despite the period of reacquisition and extinction. Inspection of Fig. 3i indicates that although a significant increase in responding on the active nose-poke at test was present (nose poke by day interaction: F(1,22) = 9.889, *p* < 0.01, $\eta_{\rho }^{2}$ = 0.31) rats in the GH (cue only) and SI (nicotine only) conditions showed robust reinstatement that was not evident in the other two groups. This conclusion is supported by a nose poke by probe test condition by housing interaction (F(1,22) = 5.194, *p* < 0.05, $\eta_{\rho }^{2}$ = 0.20). No significant differences in locomotor activity was detected (Fig. 3j).

A nicotine priming injection reinstated responding on the active nose-poke (Nose poke by day interaction: Fig. 3k; F(1,22) = 41.794, *p* < 0.001, $\eta_{\rho }^{2}$ = 0.66) to an equivalent extent in all groups. No group differences in locomotor activity were detected (Fig. 3l).

### Experiment 3: The effect of social isolation on acquisition versus reinstatement of responding for a sucrose pellet

#### Body weight

**Figure 4:**
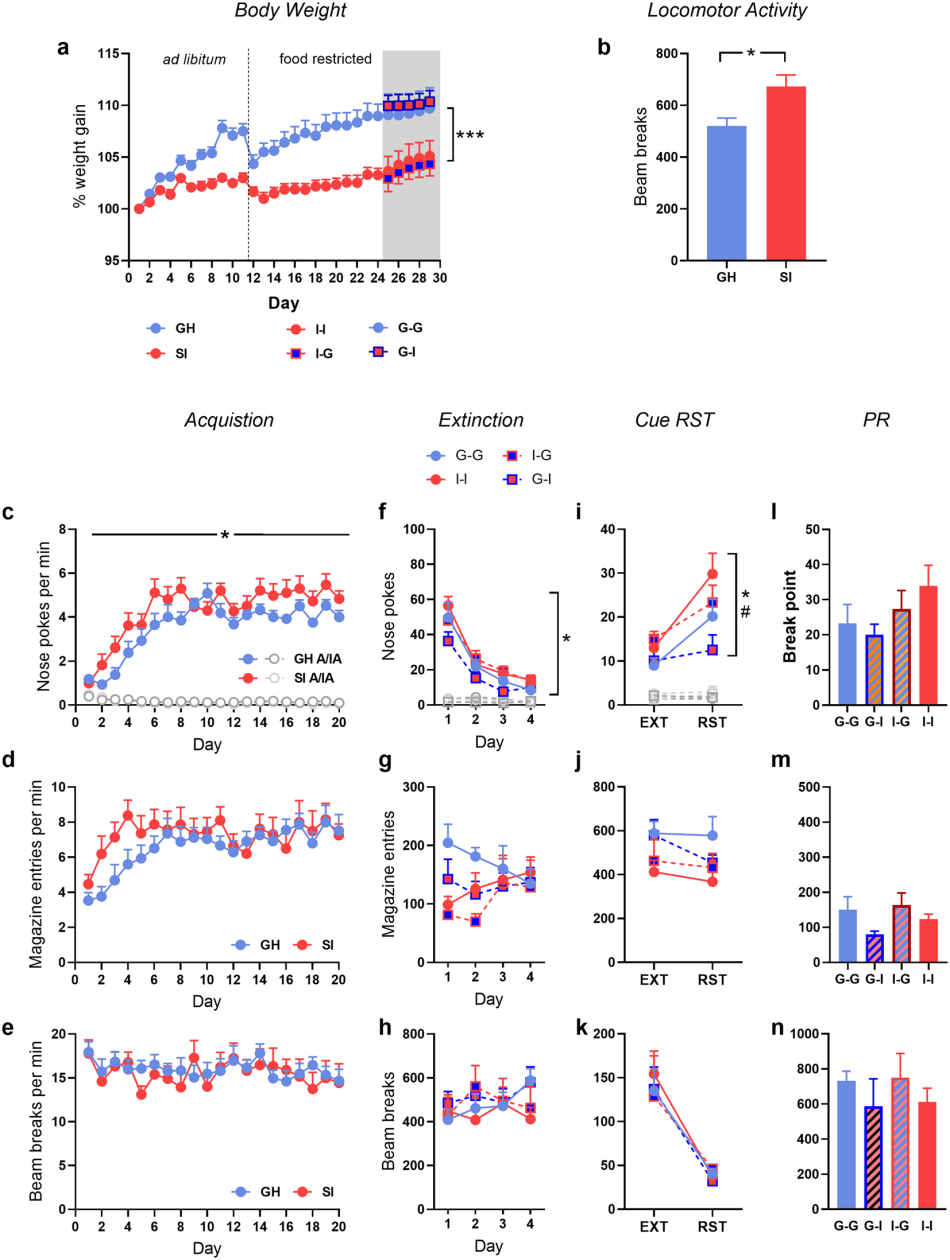
Experiment 3: The effect of reversing housing condition on acquisition versus reinstatement of responding for a sucrose pellet. (**a**) Percentage weight gain across the entire study, including when food was freely available (ad libitum), when food was restricted prior to operant training (food restricted) and with a change in housing condition (grey area). (**b**) Spontaneous locomotor activity during habituation to the instrumental chambers. Active (colour) and inactive (grey) nose-pokes, magazine entries and locomotor activity across acquisition (**c–e**), extinction training (**f–h**), cue-reinstatement (**i**–**k**), and the progressive ratio (PR) test of motivation for the food reward (**l–n**). *GH* group housed, *SI* socially isolated. *GH/G–G* group housed, *SI/I–I* socially isolated, *G–I* initially group housed, isolated after acquisition, *I–G* initially socially isolated, returned to group housing after acquisition. Symbols indicate effect or interaction including initial housing (*) or housing following the switch (#) where *p* < 0.05. n = 6–16/group.

Consistent with the previous experiments, SI rats gained less weight than their GH counterparts (Fig. 4a; main effect of housing F(1,28) = 17.69, *p* < 0.001, $\eta_{\rho }^{2}$ = 0.39; day x housing interaction: F(23,644) = 10.50, *p* < 0.001, $\eta_{\rho }^{2}$ = 0.27). This effect did not significantly change following the switch of housing condition (main effect of housing 1; F(1,26) = 9.389, *p* < 0.01, $\eta_{\rho }^{2}$ = 0.26).

#### Acquisition of instrumental conditioning

Socially isolated rats were more active during habituation to the operant chamber (Fig. 4b; F(1,29) = 7.435, *p* < 0.05, $\eta_{\rho }^{2}$ = 0.20).

As with experiment 1, rats in the SI condition more rapidly acquired nose-poking for a sucrose pellet (Fig. 4c; responses per min: main effect of housing: F(1,29) = 4.271, *p* < 0.05, $\eta_{\rho }^{2}$ = 0.13; nose poke x housing: F(1,29) = 4.829, *p* < 0.05, $\eta_{\rho }^{2}$ = 0.14), although there were no differences in magazine entries per minute (Fig. 4d) or locomotor activity per minute (Fig. 4e).

#### Extinction and reinstatement

Across extinction training rats initially social isolated (groups I–I and I–G) performed more responses on the active nose poke than those initially group housed (G–G, G–I; Fig. 4f; nose-poke x housing 1: F(1,27) = 6.33, *p* < 0.05, $\eta_{\rho }^{2}$ = 0.20), although the switch in housing condition (Housing 2) did not significantly impact on extinction. There were no differences in either magazine entries (Fig. 4g) or locomotor activity (Fig. 4h).

At the cue reinstatement test, a main effect of test day (Fig. 4i; F(1,27) = 21.274, *p* < 0.001, $\eta_{\rho }^{2}$ = 0.44) confirmed that overall, rats reinstated responding to the cue. The extent to which rats reinstated on the active nose poke depended on both the initial housing condition (Housing 1) and the post-switch housing condition (Housing 2). This was evident in a test x housing 1 × housing 2 interaction (F(1,27) = 5.046, *p* < 0.05, $\eta_{\rho }^{2}$ = 0.16) and a nose poke x test x housing 1 × housing 2 interaction (F(1,27) = 4.265, *p* < 0.05, $\eta_{\rho }^{2}$ = 0.14). The majority of these effects was driven by the initial housing condition (i.e. SI rats reinstated more), but was mitigated by the switch in housing conditions after training (i.e. SI rats that were switched to GH reinstated less). There were no significant group differences in either magazine entries (Fig. 4j) or locomotor activity (Fig. 4k).

There were no group differences in break point (Fig. 4l), magazine entries (Fig. 4m) or locomotor activity (Fig. 4n) on the progressive ratio test for motivation of reward seeking.

### Experiment 4: The effect of social isolation on acquisition versus reinstatement of responding for intravenous nicotine self-administration

#### Body weight

**Figure 5:**
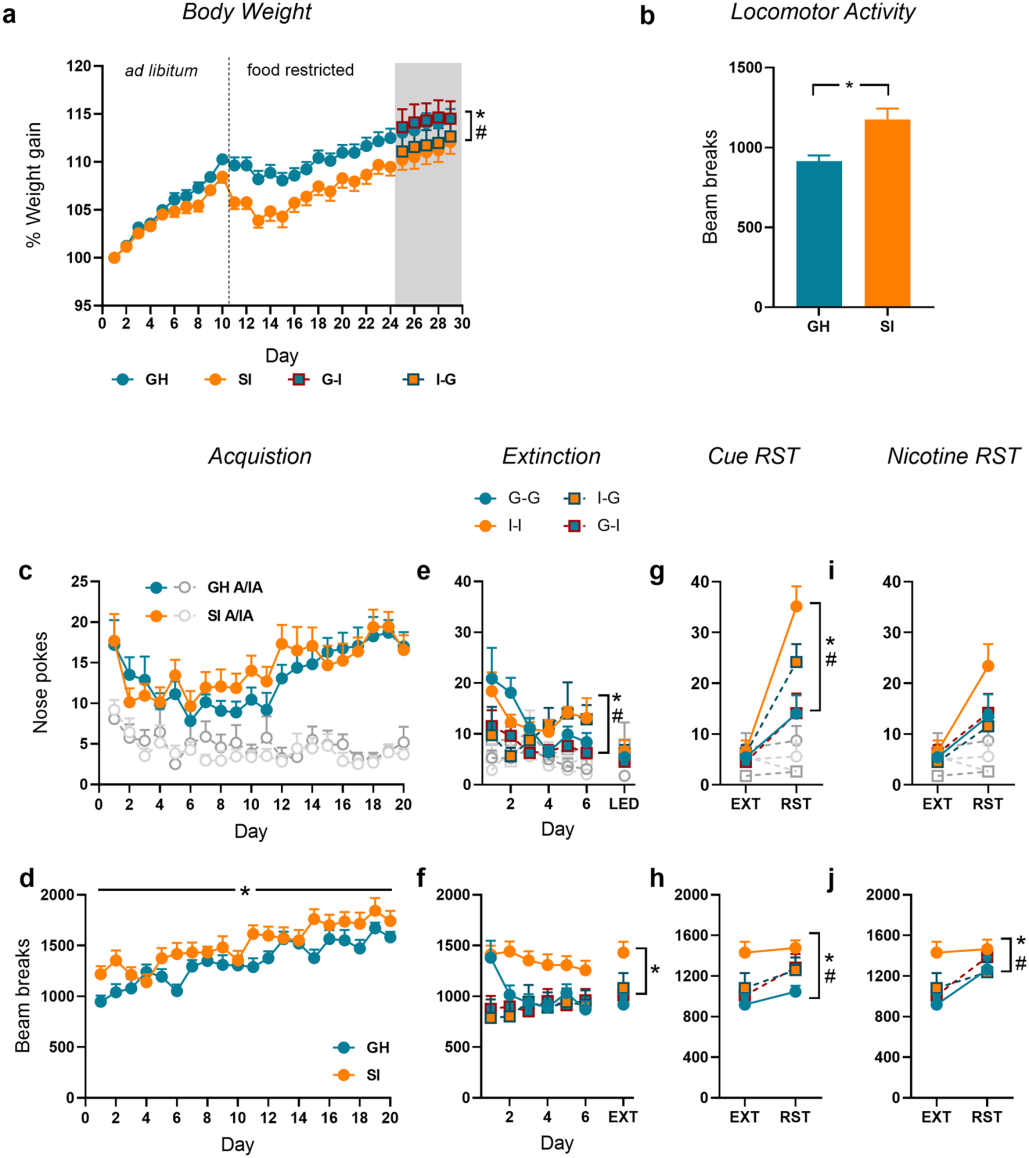
Experiment 4: The effect of reversing housing condition on acquisition versus reinstatement of responding for intravenous nicotine. (**a**) Percentage weight gain across the entire study, including when food was freely available (ad libitum), when food was restricted prior to operant training (food restricted) and with a change in housing condition (grey area). (**b**) Spontaneous locomotor activity during habituation to the instrumental chambers. Active (colour) and inactive (grey) nose-pokes and locomotor activity across acquisition (**c**, **d**), extinction training (**e**, **f**), cue-reinstatement (**h**, **i**), and nicotine reinstatement (**j**, **k**). *GH* group housed, *SI* socially isolated. *GH/G–G* group housed, *SI/I–I* socially isolated, *G–I* initially group housed, isolated after acquisition, *I–G* initially socially isolated, returned to group housing after acquisition. *LED* Last extinction day, *EXT* extinction, *RST* reinstatement. Symbols indicate effect or interaction including initial housing (*) or housing following the switch (#) where *p* < 0.05. n = 6–16/group.

As with the previous experiments, rats in the socially isolated housing condition gained less weight than group housed rats (Fig. 5a, main effect of housing: F(1,30) = 12.00, *p* < 0.01, $\eta_{\rho }^{2}$ = 0.29; day × housing 1 interaction: F(23,690) = 4.441, *p* < 0.001, $\eta_{\rho }^{2}$ = 0.13). Following the switch in housing condition, the trend of weight change across days changed as indicated by a day × group 1 × group 2 interaction (F(6,168) = 3.498, *p* < 0.01, $\eta_{\rho }^{2}$ = 0.11). Inspection of Fig. 5a suggests that this is due to a trend for decreasing body weight in the G–I rats and an increase in the I–G rats.

#### Acquisition of nicotine IV self-administration prior to switching housing conditions

Once again, socially isolated rats displayed overall higher levels of locomotor activity across habituation to the operant chamber (Fig. 5b, F(1,30) = 11.707, *p* < 0.01, $\eta_{\rho }^{2}$ = 0.28).

Housing condition did not alter the acquisition of nicotine self-administration (Fig. 5c), although SI rats displayed higher levels of locomotor activity across training (Fig. 5d; F(1,30) = 4.854, *p* < 0.05, $\eta_{\rho }^{2}$ = 0.14).

#### Extinction and reinstatement following the switch of housing condition

Across the first 6 days of extinction (minimum criterion for extinction) a Housing 1 by Housing 2 interaction (Fig. 5e; F(1,27) = 5.330, *p* < 0.05, $\eta_{\rho }^{2}$ = 0.17) indicated that the rate of responding differed depending on both initial housing, and the housing switch that occurred immediately prior to extinction training. However, this difference rapidly dissipated, as the number of days taken to achieve the extinction criterion (G–G = 13.13 ± 0.52; G–I = 11.75 ± 0.70; I–G = 12.88 ± 0.70; I–I = 13.14 ± 0.70) and responding on the last day of extinction training did not differ between groups (Fig. 5e: EXT).

Locomotor activity across the first days of extinction training was influenced both by Housing 1 and Housing 2 (day × housing 1 × housing 2 interaction: F(5,135) = 3.879, *p* < 0.01, $\eta_{\rho }^{2}$ = 0.29). However inspection of Fig. 5f, demonstrates that a persistent difference in activity was only evident in group I–I, and that this difference remained on the final day of extinction training (main effect of housing 1; F(1,24) = 5.801, *p* < 0.05, $\eta_{\rho }^{2}$ = 0.20).

A main effect of test (F(1,24) = 49.224, *p* < 0.001, $\eta_{\rho }^{2}$ = 0.67) indicated that overall, rats reinstated responding to the cue (Fig. 5g). Comparison of responding across cue-induced reinstatement revealed that again, socially isolated rats demonstrated higher levels of cue-induced reinstatement (main effect of housing 1: F(1,24) = 9.116, *p* < 0.05, $\eta_{\rho }^{2}$ = 0.27) and that this was mitigated by a return to group housing (housing 1 by housing 2 interaction: F(1,24) = 5.866, *p* < 0.05, $\eta_{\rho }^{2}$ = 0.20).

Locomotor activity increased slightly with test (Fig. 5h; F(1,24) = 5.547, *p* < 0.05, $\eta_{\rho }^{2}$ = 0.19) and a main effect of both housing 1 (F(1,24) = 7.379, *p* < 0.05, $\eta_{\rho }^{2}$ = 0.24) and housing 2 (F(1,24) = 4.806, *p* < 0.05, $\eta_{\rho }^{2}$ = 0.17) reflected the overall higher levels of activity in the I–I rats, and comparable levels of activity in G–I and I–G rats.

Following a nicotine priming injection, all rats reinstated responding (Fig. 5i; F(1,24) = 17.232, *p* < 0.001, $\eta_{\rho }^{2}$ = 0.38), and although strong trends for an effect of housing were evident (*p* > 0.06), no group differences were detected. An increase in locomotor activity was detected following the nicotine prime (Fig. 5j), and this was greater in the initially group housed animals (day x housing 1: F(1,25) = 4.637, *p* < 0.05, $\eta_{\rho }^{2}$ = 0.16), but was overall influenced by housing 2 independently of housing 1 (main effect of housing 2: F(1,25) = 4.931, *p* < 0.05, $\eta_{\rho }^{2}$ = 0.17).

## Discussion

Social isolation in rats has a marked impact on rat physiology and reward-seeking. Socially isolated rats showed elevated baseline CORT, gained significantly less weight across the study, were more active in response to a novel or familiar environment, acquired nose-poking for a food pellet more rapidly, and showed increased susceptibility to cue-, but not reward-induced reinstatement. Notably, these effects are partially mitigated by a return to group housing, suggesting that they are not necessarily permanent, and that a return to a social setting can quickly reverse any deficits or changes associated with social isolation.

Enhanced acquisition of drug-seeking in socially isolated rats has been reported previously with heroin^30^ and cocaine^31^, although this frequently involves isolation during the early post-natal period^40^ or adolescence^41^. Here we show that although 20 days of isolation in adult rats has a minor effect on the acquisition of operant responding (particularly for food pellets), isolation may change the way rats are learning about response-contingent and reward associated cues across this period, particularly for nicotine. Across the probe sessions where rats were allocated to receive either nicotine or response-contingent cues, rats in the group housed condition responded more for the response-contingent cues than for nicotine alone, confirming the ability of nicotine to act as a potent reinforcement enhancer^42^ and confirming the importance of nicotine-associated cues in maintaining nicotine seeking^43^.

In contrast, socially isolated rats responded at an equivalent level for either nicotine, or its previously paired cues, indicating one of two possibilities. The first is that socially isolated rats attribute equal value to nicotine and the cues that accompany its infusion, and the presence of either of these outcomes is sufficient to maintain responding^43,44^. The second possibility is that rats are inflexible in their behaviour, nose-poking as a consequence of the drug-paired context, and independently of the current value of the reward (i.e. habitual). The ability of social isolation to promote the development of habits has been reported previously^45^, however further research is required to distinguish between these two competing possibilities.

Further evidence for an increased role of reward-associated cues in reward-seeking of socially isolated rats comes from the reinstatement tests. Whereas the probe sessions tested the ability of cues to maintain an established response, reinstatement tests assess the ability of the extinguished response to be reinstated, which has been shown to involve different circuitry within the brain^46^. Under these conditions, the cue was more potent in reinstating responding for both a food and nicotine reward in socially isolated rats, indicating that social isolation has potentiated the conditioned reinforcing properties of the reward-paired cue. Notably, there were no differences in responding in reward-primed reinstatement, suggesting that the reward itself was equally valued across housing conditions. This pattern of results is largely consistent with previous literature, where both social isolation or an impoverished physical environment have been shown to enhance cue-induced reinstatement of cocaine seeking, with little impact on drug reinstatement^34,35,47^. It suggests that social isolation enhances the encoding of reward-related cues across acquisition of both nicotine and sucrose self-administration, and that following extinction, these cues are more readily able to reinstate reward seeking.

We next aimed to determine whether the impact of social isolation on the ability of the cue to motivate responding was reversible with a return to group housing, and at the same time determine if the difference in cue-reinstatement was due to alteration in how the cues were encoded across acquisition, or how readily they are retrieved at test. To achieve this, housing condition was switched in half of the animals after training and before extinction and testing. Here we found that returning socially isolated rats to group housing partially reversed the impact of social isolation on sensitivity to cue-induced relapse for both food and nicotine rewards. This effect is consistent with the environmental enrichment literature, where the introduction of environmental enrichment after training can reduce cue-induced reinstatement across a range of drug classes^47,48^, including nicotine^32^. It supports the idea that introduction of social enrichment may help to mitigate craving in already established drug users.

In contrast, switching from group housing across acquisition to isolation at test did not enhance relapse. In this latter condition, our data would suggest that a switch from group to socially isolated housing should enhance cue reinstatement, and this was not observed here either with the pellet or nicotine reward. Although further studies are required to explore this result, it may have been due to the relatively short period of isolation prior to testing (4–6 d) compared to the I–I or I–G groups (20 d isolation) that was not sufficient to impact on behaviour. Alternatively, it may suggest that the dominant driver of responding at reinstatement is the influence of information encoded across acquisition (when group housed), and this has a lasting influence over performance at test, and that group housing during training produces a more robust phenotype. Together this data indicates that the source of difference in the cue-reinstatement is due to both the encoding of the reward-cue associations across acquisition (I–I and I–G rats > G–G and G–I rats reinstatement at test), but also the retrieval and expression of this information at test (I–I > I–G).

The finding that altered encoding and retrieval of reward-associated cues in adult rats is reversible, is particularly relevant considering that many studies have shown an array of molecular changes in response to social isolation in rodents, ranging from alterations in stress-related neuropeptides^49^ and neurotransmitter signalling^50^ to loss of myelination^51^. Our data suggests that the impact of social isolation on reward-related behaviour is transient in adult rats and can be at least partially reversed with a return to group housing. Whether the same follows for these molecular markers remains to be determined, but may have consequences for the treatment of substance abuse disorders, where reengaging with social groups may be just as beneficial as pharmacological interventions^36^.

It is worth noting that beyond the described impact on cue-learning, social isolation produced significant impacts on the rat physiology. Socially isolated rats displayed high CORT, reflecting elevated baseline levels of circulating stress hormones as has been reported previously^23,33^. These rats gained much less weight than those group housed, despite equivalent food intake (all rats ate all of their food allocation within a 24-h period) and motivation for food (PR test and pellet consumption). This may be due to metabolic changes in these rats due to isolation (i.e. more energy expenditure for heating, higher activity) or as a consequence of circulating levels of stress hormones^52^. Irrespective of the cause, it is clear that social isolation of these rats (even in the presence of an enriched environment), has profound effects on rats physiology that extend beyond the psychological processes that were the focus of this study.

The wide-ranging changes in behaviour and physiology reported here are particularly relevant when considering the frequency with which animals are socially isolated across a range of laboratory settings. Although this commonly occurs by necessity in studies of feeding, it also prevalent in drug self-administration and may account for inconsistencies in data from different labs. For example, where animals in Australasia^44,53^ are typically group housed, in many other laboratories, rats are pair housed^54^ and, rats in North America are typically individually housed either prior to, or immediately after surgery^55,56^. It is perhaps not surprising then that the rates of cue-induced self-administration are markedly higher in those studies where rats are isolated (e.g. compare^56^ vs.^57^). While numerous other variables exist, this difference may have a flow on effect to studies of the mechanisms underlying drug addiction. Although certainly, drug addiction in humans occurs under circumstances of, or as a consequence of, social isolation, when modelling and assessing relapse, social isolation of what is otherwise a social animal, should be considered and justified appropriately.

In conclusion, our data suggest that social isolation of rats produces changes in how animals encode and retrieve information about reward-associated cues in their environment and then use these cues as conditioned reinforcers to motivate responding. Importantly, these changes can be reversed with a return to group housing, suggesting that the behavioural mechanisms underlying this phenomenon are flexible. Understanding how social isolation impacts on reward-processing may allow for a more specific and tailored approach to treatment for those living alone (i.e. cue exposure therapy) versus those living in a social setting.

## Data Availability

All data is available on request.
